# Supplementary material for: Modeling glioblastoma heterogeneity as a dynamic network of cell states
Source: Mol Syst Biol. 2021 Sep 16;17(9):e10105. doi: 10.15252/msb.202010105 (PMC8444284; doi:10.15252/msb.202010105)
Supplement: Supplementary file 6 — Source Data for Figure 5 [file MSB-17-e10105-s004.zip › Figure5A_sourcedata/GSEA_3071/hallmarks_stateVI.GseaPreranked.1623742557031/HALLMARK_IL2_STAT5_SIGNALING.html]

Details for gene set HALLMARK\_IL2\_STAT5\_SIGNALING[GSEA]

|  || Dataset | state63071 |
| Phenotype | NoPhenotypeAvailable |
| Upregulated in class | na\_pos |
| GeneSet | HALLMARK\_IL2\_STAT5\_SIGNALING |
| Enrichment Score (ES) | 0.24502885 |
| Normalized Enrichment Score (NES) | 1.6279037 |
| Nominal p-value | 0.005988024 |
| FDR q-value | 0.04506621 |
| FWER p-Value | 0.186 |
Table: GSEA Results Summary

  

Fig 1: Enrichment plot: HALLMARK\_IL2\_STAT5\_SIGNALING      
 Profile of the Running ES Score & Positions of GeneSet Members on the Rank Ordered List

  

| PROBE | GENE SYMBOL | GENE\_TITLE | RANK IN GENE LIST | RANK METRIC SCORE | RUNNING ES | CORE ENRICHMENT || 1 | TGM2 |  |  | 3 | 1.367 | 0.0414 | Yes |
| 2 | ALCAM |  |  | 7 | 1.185 | 0.0772 | Yes |
| 3 | ODC1 |  |  | 38 | 0.918 | 0.0972 | Yes |
| 4 | MYC |  |  | 69 | 0.815 | 0.1141 | Yes |
| 5 | SOCS2 |  |  | 72 | 0.803 | 0.1383 | Yes |
| 6 | HOPX |  |  | 89 | 0.761 | 0.1574 | Yes |
| 7 | GADD45B |  |  | 90 | 0.758 | 0.1808 | Yes |
| 8 | AHNAK |  |  | 139 | 0.680 | 0.1885 | Yes |
| 9 | BCL2L1 |  |  | 247 | 0.567 | 0.1761 | Yes |
| 10 | MAFF |  |  | 253 | 0.562 | 0.1920 | Yes |
| 11 | XBP1 |  |  | 389 | 0.494 | 0.1695 | Yes |
| 12 | PLAGL1 |  |  | 400 | 0.490 | 0.1819 | Yes |
| 13 | SOCS1 |  |  | 489 | 0.458 | 0.1715 | Yes |
| 14 | CKAP4 |  |  | 497 | 0.456 | 0.1836 | Yes |
| 15 | PIM1 |  |  | 582 | 0.430 | 0.1734 | Yes |
| 16 | SERPINB6 |  |  | 583 | 0.430 | 0.1867 | Yes |
| 17 | PNP |  |  | 625 | 0.418 | 0.1882 | Yes |
| 18 | TNFSF10 |  |  | 631 | 0.416 | 0.1997 | Yes |
| 19 | ARL4A |  |  | 704 | 0.395 | 0.1918 | Yes |
| 20 | NT5E |  |  | 787 | 0.379 | 0.1806 | Yes |
| 21 | NOP2 |  |  | 821 | 0.373 | 0.1829 | Yes |
| 22 | CAPG |  |  | 851 | 0.368 | 0.1861 | Yes |
| 23 | KLF6 |  |  | 854 | 0.368 | 0.1970 | Yes |
| 24 | GSTO1 |  |  | 871 | 0.365 | 0.2038 | Yes |
| 25 | PUS1 |  |  | 933 | 0.355 | 0.1977 | Yes |
| 26 | BMP2 |  |  | 940 | 0.354 | 0.2070 | Yes |
| 27 | NFIL3 |  |  | 956 | 0.352 | 0.2137 | Yes |
| 28 | PTRH2 |  |  | 1020 | 0.341 | 0.2066 | Yes |
| 29 | TRAF1 |  |  | 1040 | 0.339 | 0.2118 | Yes |
| 30 | RNH1 |  |  | 1056 | 0.336 | 0.2180 | Yes |
| 31 | MYO1C |  |  | 1059 | 0.336 | 0.2278 | Yes |
| 32 | CCND2 |  |  | 1064 | 0.335 | 0.2370 | Yes |
| 33 | FAH |  |  | 1082 | 0.333 | 0.2426 | Yes |
| 34 | SLC1A5 |  |  | 1169 | 0.320 | 0.2284 | Yes |
| 35 | PDCD2L |  |  | 1181 | 0.318 | 0.2352 | Yes |
| 36 | GALM |  |  | 1182 | 0.318 | 0.2450 | Yes |
| 37 | GLIPR2 |  |  | 1377 | 0.294 | 0.1999 | No |
| 38 | ENPP1 |  |  | 1677 | 0.262 | 0.1243 | No |
| 39 | FURIN |  |  | 1702 | 0.260 | 0.1257 | No |
| 40 | LIF |  |  | 1804 | 0.250 | 0.1051 | No |
| 41 | PRNP |  |  | 1866 | -0.260 | 0.0961 | No |
| 42 | APLP1 |  |  | 2259 | -0.325 | -0.0035 | No |
| 43 | ITGA6 |  |  | 2267 | -0.327 | 0.0047 | No |
| 44 | TIAM1 |  |  | 2303 | -0.334 | 0.0052 | No |
| 45 | ECM1 |  |  | 2309 | -0.334 | 0.0142 | No |
| 46 | DENND5A |  |  | 2332 | -0.339 | 0.0185 | No |
| 47 | PHTF2 |  |  | 2382 | -0.350 | 0.0156 | No |
| 48 | ITGAV |  |  | 2476 | -0.371 | 0.0011 | No |
| 49 | ST3GAL4 |  |  | 2604 | -0.402 | -0.0220 | No |
| 50 | IGF1R |  |  | 2618 | -0.404 | -0.0131 | No |
| 51 | CASP3 |  |  | 2699 | -0.426 | -0.0223 | No |
| 52 | IGF2R |  |  | 2838 | -0.469 | -0.0464 | No |
| 53 | IFITM3 |  |  | 2851 | -0.477 | -0.0351 | No |
| 54 | ITIH5 |  |  | 2874 | -0.485 | -0.0262 | No |
| 55 | POU2F1 |  |  | 2908 | -0.498 | -0.0200 | No |
| 56 | GPX4 |  |  | 2913 | -0.499 | -0.0057 | No |
| 57 | BMPR2 |  |  | 2930 | -0.506 | 0.0055 | No |
| 58 | CDC6 |  |  | 2990 | -0.534 | 0.0055 | No |
| 59 | CD81 |  |  | 3056 | -0.566 | 0.0048 | No |
| 60 | TNFRSF21 |  |  | 3125 | -0.600 | 0.0043 | No |
| 61 | RGS16 |  |  | 3304 | -0.765 | -0.0218 | No |
| 62 | SPRY4 |  |  | 3325 | -0.793 | -0.0029 | No |
| 63 | SPP1 |  |  | 3541 | -1.286 | -0.0232 | No |
| 64 | SYT11 |  |  | 3587 | -1.620 | 0.0143 | No |
Table: GSEA details [plain text format]

  

Fig 2: HALLMARK\_IL2\_STAT5\_SIGNALING: Random ES distribution      
 Gene set null distribution of ES for **HALLMARK\_IL2\_STAT5\_SIGNALING**
